# Photo-plasmonic effect as the hot electron generation mechanism

**DOI:** 10.1038/s41598-023-27775-1

**Published:** 2023-01-11

**Authors:** M. Akbari-Moghanjoughi

**Affiliations:** Faculty of Sciences, Department of Physics, Azarbaijan Shahid Madani University, 51745-406 Tabriz, Iran

**Keywords:** Fluid dynamics, Plasma physics, Quantum physics

## Abstract

Based on the effective Schrödinger–Poisson model a new physical mechanism for resonant hot-electron generation at irradiated half-space metal–vacuum interface of electron gas with arbitrary degree of degeneracy is proposed. The energy dispersion of undamped plasmons in the coupled Hermitian Schrödinger–Poisson system reveals an exceptional point coinciding the minimum energy of plasmon conduction band. Existence of such exceptional behavior is a well-know character of damped oscillation which in this case refers to resonant wave–particle interactions analogous to the collisionless Landau damping effect. The damped Schrödinger–Poisson system is used to model the collective electron tunneling into the vacuum. The damped plasmon energy dispersion is shown to have a full-featured exceptional point structure with variety of interesting technological applications. In the band gap of the damped collective excitation,depending on the tunneling parameter value, there is a resonant energy orbital for which the wave-like growing of collective excitations cancels the damping of the single electron tunneling wavefunction. This important feature is solely due to dual-tone wave-particle oscillations, characteristics of the collective excitations in the quantum electron system leading to a resonant photo-plasmonic effect, as a collective analog of the well-known photo-electric effect. The few nanometer wavelengths high-energy collective photo-electrons emanating from the metallic surfaces can lead to a much higher efficiency of plasmonic solar cell devices, as compared to their semiconductor counterpart of electron–hole excitations at the Fermi energy level. The photo-plasmonic effect may also be used to study the quantum electron tunneling and electron spill-out at metallic surfaces. Current findings may help to design more efficient spasers by using the feature-rich plasmonic exceptional point structure.

## Introduction

Free electrons in metals and semiconductors control almost all fundamental physical properties of the solid^1^. Some basic properties of metals such as optical, electronic and thermodynamic ones are quite satisfactorily described by noninteracting electron (Drude) model^2^. The band structure model, on the other hand, has been a huge success and advancement in understanding of important quantum mechanical features of semiconductor material which was urgent in consequent development of the cutting edge nanoelectronic technology^3,4^. There are variety of collective aspects of the electron gas with fundamental applications in plasmonics^5–7^, optoelectronics^8,9^, nanotechnology^10^ etc., which require advanced quantum many-body theories to cope with^11,12^. Quantum electrodynamic^13^, density function^14^ and quantum hydrodynamic^15–21^ theories have been some of the tools of the advanced solid state theory in order to describe collective physical behavior of quantum electron gas. Quantum kinetic and hydrodynamic theories are however among more common tools in the study of collective plasmonic excitations in more complex plasmas where many charged species are involved. These models are the results of previous pioneering works on collective quantum electron excitation theories^22–38^. There has been also many recent advancements in development of hydrodynamic theories and their successful employment in investigations of linear and nonlinear aspects of collective phenomena in dense degenerate plasmas involved both in the laboratory scale phenomena^39–52^ as well as of the astrophysical scale^53–58^. One of the oldest descendent of the quantum hydrodynamic theory is pioneered by Madelung^59^, referred to as the quantum fluid theory. The extended many-body version of the later theory, i.e. effective Schrödinger–Poisson model^60^, has shown some success in the study of key features of collective properties of the quantum electron gas. Among those one may call the calculation of generalized energy band structure of a multistream electron system^61^, quantization of collective electron gas excitations^62^, collective effects on the electronic heat capacity in metals^63^, edge plasmon excitations and electron spill out effect^64^, dual-plasmon scattering^65^, collective quantum interference^66^, etc.^67–69^.

Plasmonics is one of the technologically appealing interdisciplinary active fields of physical sciences with a vigorous development over the past 2 decades^70,71^. With the turn of the millennium the plasmonic field has not achieved the desired pace with the rapidly growing semiconductor industry of solar cell^72^, sensor^73^, modulator^74^ and communications device^75^ technologies for some definite reasons. Collective electron energy extraction from fast response plasmonic devices requires elimination of damping effects which is inherent in typical metals due to effects such as the electron–phonon scattering and low mean free path collective response. While the electron–electron scattering is greatly inhibited at high electron densities due to the Pauli blocking mechanism, the reduction of electron–phonon scattering effect is costly due to atomic structure design and perfect crystal growth. Even in the absence of electron–ion collisions kinetic effects such as the collisionless Landau damping substantially limit the plasmonic device operation. Moreover, quantum charge screening in metals inhibits the long-range collective response of plasmonic devices. However, if the known technological obstacles is overcome, plasmonic devices can find new applications in optical emitters^76^, plasmon focusing^70^, nanoscale waveguiding^77^ and optical antennas^78^, nanoscale swiches^79^ and plasmonic lasers^80^. Plasmon excitations may become highly efficient means of effective solar energy conversion towards improved photovoltaic and catalytic designs by collective energy transport. However, the new plasmonic energy conversion may require several considerations regarding the materials, architecture and fabrication methods^81,82^. Recent investigation shows that guiding and concentration of light using plasmonic geometries leads to entirely new solar-cell designs in which light is more efficiently absorbed in a single quantum well, or in a single layer of quantum dots or molecular chromophores^83^. Plasmonic devices may operate at small wavelengths in the range (10–380) nm in high precision engineering design with relatively higher cost of design. The generated energetic electrons can be collected in a tandem design with an appropriate electron accepting nanolayer such as, TiO$_2$^84^. Due to vast applications and technological appeal, there has been an ongoing intense research towards exploration of plasmonic field in recent years^85–90^.

One of the great advantages of plasmonic technology is the parametric control over the collective effects such as excitation instabilities by means of exceptional point structure. Due to both wave- and particle-like dual oscillations in plasmons, a lossless energy exchange among wave and single-electron behavior may be possible and used to manipulate the physical properties of the quantum plasmonic devices. These singular exceptional points are critical points around which the physical behavior of the system changes radically. Exceptional points has been used in parametric control of the behavior of various coupled physical systems such as mechanical, optical, quantum, etc.^91^. In current research we show that the plasmonic system processes a full-featured exceptional point phase structure with variety of possible applications in plasmonic and semiconductor device fabrication. In this work we also introduce a feasible photo-plasmonic mechanism for energetic electron tunneling through the metal surface based on the exceptional point theory of the damped Schrödinger–Poisson system. The arrangement of the paper is as follows. We present the theoretical model in “The theoretical model”. In “Plasmon dispersion and exceptional point structure”, we describe the exceptional point structure of the lossless Hermitian system. The non-Hermitian system as a model for half-space plasmon excitations is presented in “The pseudo-damped plasmon excitations”. The photo-plasmonic process via the collective electron tunneling is introduced in ”Half-space excitations and photo-plasmonic effect” and conclusion is drawn in “Conclusion”.

## The theoretical model

We consider electron gas of arbitrary degeneracy with a neutralizing positive ionic background. The dynamics of the quantum electron fluid may be described using the following effective Schrödinger–Poisson system^92^

1a $$\begin{aligned}&i\hbar \frac{{\partial \mathcal{{N}}(\mathbf{{r}},t)}}{{\partial t}} = - \frac{{{\hbar ^2}}}{{2m}}\Delta \mathcal{{N}}(\mathbf{{r}},t) - e\phi (\mathbf{{r}})\mathcal{{N}}(\mathbf{{r}},t) + \mu \mathcal{{N}}(\mathbf{{r}},t), \end{aligned}$$

1b $$\begin{aligned}&\Delta \phi (\mathbf{{r}}) = 4\pi e (|\mathcal{{N}}(\mathbf{{r}},t)|^2-n_0), \end{aligned}$$

where $\mathcal{{N}}(\mathbf{{r}},t)=\psi (\mathbf{{r}},t)\exp [iS(\mathbf{{r}},t)/\hbar ]$ is the statefunction characterizing the probability density of the system which is related to the local electron number density $n(\mathbf{{r}})=\psi (\mathbf{{r}})\psi ^*(\mathbf{{r}})$ with $n_0$ being the positive background density and the electron fluid momentum is given by $\mathbf{{p}}({\mathbf{{r}},t)}=\nabla {S(\mathbf{{r}},t)}$. The chemical potential $\mu$ is related to the local electron number density and the equilibrium temperature by the following isothermal equation of state (EoS)

2a $$\begin{aligned}&{n(\mu _0,T)} = \frac{{{2^{1/2}}m{^{3/2}}}}{{{\pi ^2}{\hbar ^3}}} \int _{0}^{ + \infty } {\frac{{\sqrt{{\epsilon }} d{\epsilon }}}{{{e^{\beta ({\epsilon }-\mu _0)}} + 1}}}, \end{aligned}$$

2b $$\begin{aligned}&{P(\mu _0,T)} = \frac{{{2^{3/2}} m{^{3/2}}}}{{3{\pi ^2}{\hbar ^3}}}\int _0^{ + \infty } {\frac{{{{\epsilon }^{3/2}} d{\epsilon }}}{{{e^{\beta ({\epsilon } - {\mu _0})}} + 1}}.} \end{aligned}$$

where $\beta =\mu _0/k_B T$ with *T* being the electron temperature and *P* the quantum statistical pressure. Note that the thermodynamic identity $n\nabla \mu =\nabla P(n)$ is satisfied by the EoS (2).

Assuming near-equilibrium perturbations in the static limit $\mathbf{{p}}=0$ with separable solutions of type $\psi (\mathbf{{r}},t)=\psi (\mathbf{{r}})\psi (t)$, one may appropriately linearize the coupled system around the equilibrium collective quantum-state, $\{\psi ^0=1,\phi ^0=0,\mu ^0=\mu _0\}$, in order to obtain the following normalized coupled system

3a $$\begin{aligned}&{i}{\hbar }\frac{{\partial \psi (t)}}{{\partial t}} = \epsilon \psi (t), \end{aligned}$$

3b $$\begin{aligned}&\Delta \Psi (\mathbf{{r}}) + \Phi (\mathbf{{r}}) = - E\Psi (\mathbf{{r}}), \end{aligned}$$

3c $$\begin{aligned}&\Delta \Phi (\mathbf{{r}}) - \Psi (\mathbf{{r}}) = 0, \end{aligned}$$

where $\Psi (\mathbf{{r}})=\psi (\mathbf{{r}})/n_0$ and $\Phi (\mathbf{{r}})=e\phi (\mathbf{{r}})/E_p$ are the normalized local number density and electrostatic energy functions characterizing the arbitrary degenerate electron gas excitations. The energy eigenvalue of the system has been defined as $E=(\epsilon -\mu _0)/E_p$ which is the energy eigenvalues $\epsilon$ measured relative to the Fermi energy level and normalized to the plasmon energy $E_p=\hbar \omega _p$ with $\omega _p=\sqrt{4\pi e^2 n_0/m}$ being the plasmon frequency. Note that the space and time variables in equation set (5) are scaled by the plasmon length $l_p=1/k_p$ with $k_p=\sqrt{2m E_p}/\hbar$ being the characteristic plasmon wavenumber and inverse plasmon frequency, respectively.

The Fourier analysis of the system (5) leads to the generalized matter-wave energy dispersion of $E=k_w^2+k_e^2$ where $k_w$ and $k_e$ characterize the wave-like and particle-like oscillations, respectively. The dual wavenumber character of oscillations is an intrinsic feature of quantum electron fluid excitations^65^. Moreover, the coupled wave-particle oscillations admit a complementarity-like relation $k_w k_e=1$ in dimensionless form. The energy dispersion can be written in a more useful form of $E=k^2+1/k^2$ in which *k* is the characteristic wavenumber of collective electron excitations.

Using the standard quantum statistical definitions and the general energy dispersion for undamped plasmons (5), one may obtain thermodynamic quantities for collective excitations in a homogenous arbitrary degenerate electron gas. Since there is a one-to-one correspondence between the single-electron and collective excitations energy levels^62^, analogous to quantum liquid quasiparticle states, the Pauli exclusion principle also applies to collective modes. Starting with the number of quasiparticle modes we have $N(k)=4\pi k^3/3$ which leads to the density of states (DoS) $D(k)=(dN/dk)/|dE/dk|$, given the quasiparticle occupation function of $F(k,\theta )=1/[1+\exp (E/\theta )]$, in which $\theta =T/T_p$ is the normalized electron temperature to the plasmon temperature defined as, $T_p=E_p/k_B$. Some important quantities such as the normalized number-density $n(\theta )$, internal energy $U(\theta )$ and plasmon heat capacity $c(\theta )$ are given as

4a $$\begin{aligned}&n(\theta ) = \int \limits _0^\infty {\frac{{{D(k)}{d_k}{E(k)}dk}}{{1 + \exp ({E}/\theta )}}},\quad {D(k)} = \frac{{2\pi {k^5}}}{{\left| {{k^4} - 1} \right| }}, \end{aligned}$$

4b $$\begin{aligned}&U(\theta ) = \int \limits _0^\infty {\frac{{{D(k)}{E(k)}{d_k}{E(k)}dk}}{{1 + \exp ({E}/\theta )}}},\quad {E(k)} = {k^2} + \frac{1}{{{k^2}}}, \end{aligned}$$

4c $$\begin{aligned}{}&c(\theta ) = \frac{1}{{4{\theta ^2}}}\int \limits _0^\infty D(k){E^2}(k){d_k}E(k){\mathop{\textrm{sech}}} (E/2\theta )dk, \end{aligned}$$

**Figure 1:**
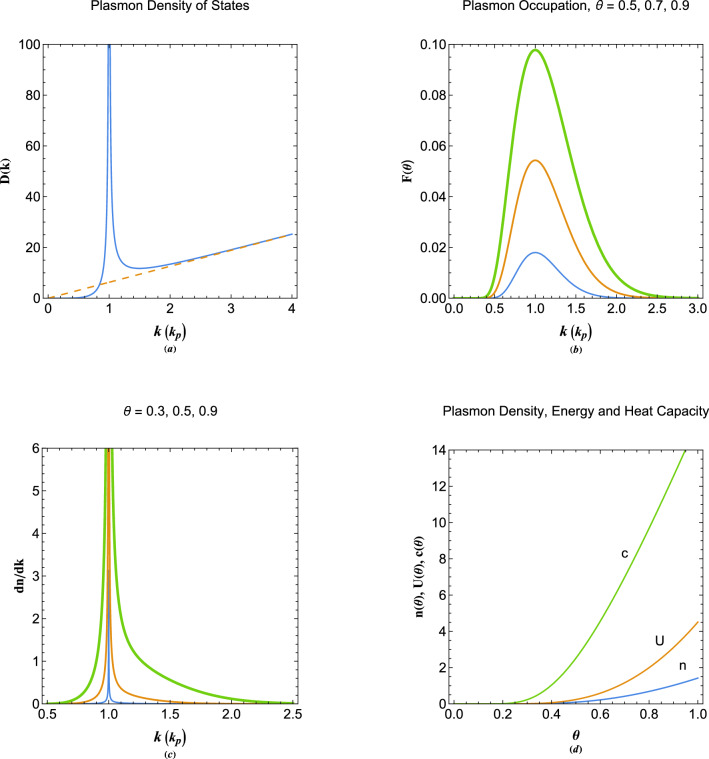
(**a**) The normalized plasmon density of states (DoS) as a function of the plasmon wavenumber showing a Van Hove-like singularity at the plasmon surface. (**b**) The normalized plasmon occupation function variation with the wavenumber for different normalized electron temperature. The increase in the curve thickness indicate the increase in the electron temperature. (**c**) The normalized quasiparticle mode per wavenumber for different electron temperature values. (**d**) The plasmon quasiparticle number-density, internal energy and heat capacity as a function of the electron temperature.

Figure 1 depicts thermodynamic properties of collective excitations (quasiparticles) in the electron gas of arbitrary degeneracy. On the label of horizontal wavenumber axis in many plots the characteristic plasmon wavenumber appears within parenthesis denoting the normalization unit of the corresponding axes. Figure 1a depicts the quasiparticle DoS as a function of wavenumber. A Van-Hove-like singularity is present at the critical point $k=k_p$ on the plasmon surface, analogous to the similar singularity at the Fermi surface of crystalline solid^1^. It is remarked that DoS increases with increase of plasmon wavenumber almost linearly for large wavenumber values, quite similar to the case of free-electron gas^1^. The plasmon occupation function is shown in Fig. 1b for different values of normalized electron temperature. The plasmon quasiparticle occupation function reveals a distinct difference with that of the free electron model. It is remarked that occupation of the collective modes at the long wavelength limit is strongly lowered, as opposed to the free electron model^1^. It is also shown that increase of the normalized electron temperature leads to overall increase in occupation probability of quasiparticles at wavenumbers close to the plasmon wavenumber. In Fig. 1c, we show the quasiparticle mode per wavenumber, $D(k) F(k,\theta )$. The largest mode per wavenumber are located around the plasmon wavenumber and increases with increase in normalized electron temperature. However, this increase is seen to be more significant for $k>k_p$ as compared to $k<k_p$. The normalized quasiparticle number-density, internal energy and heat capacity due to collective electron excitations as a function of the normalized electron temperature are shown in Fig. 1d. It is remarked that the number-density, internal energy and heat capacity of quasiparticles increase rapidly with increase of the normalized electron temperature, $\theta$.

## Plasmon dispersion and exceptional point structure

The 3D linearized system (5) has been solved exactly in stationary radial form in Ref.^66^. However, the one dimensional case, which admits simple stationary analytic solution with many applications, is considered here. The time-independent 1D linearized coupled system of interest is^65^

5a $$\begin{aligned}&\frac{{d^2{\Psi (x)}}}{{d{x^2}}} + \Phi (x) + E\Psi (x) = 0, \end{aligned}$$

5b $$\begin{aligned}&\frac{{d^2{\Phi (x)}}}{{d{x^2}}} - {\Psi }(x) = 0, \end{aligned}$$

where $\Psi (x)\Psi ^*(x)=n(x)$ and $\Phi (x)$ denotes the local electrostatic potential energy. Assuming the boundary values $\Psi (0)=1$ ans $\Phi (0)=\Psi '(0)=\Phi '(0)=0$, we have the solution of the form

6a $$\begin{aligned}&\Phi (x) = \frac{{\cos ({k_w}x) - \cos ({k_e}x)}}{{\sqrt{{{E}^2} - 4} }}, \end{aligned}$$

6b $$\begin{aligned}&\Psi (x) = \frac{{\left( {{E} + \sqrt{{{E}^2} - 4} } \right) \cos ({k_e}x) - \left( {{E} - \sqrt{{{E}^2} - 4} } \right) \cos ({k_w}x)}}{{2\sqrt{{{E}^2} - 4} }}, \end{aligned}$$

where $k_w$ and $k_e$ are wave- and particle-like wavenumbers of the coupled oscillations

7 $$\begin{aligned} {k_w} = \sqrt{\frac{{{E} - \sqrt{{{E}^2} - 4} }}{2}},\quad {k_e} = \sqrt{\frac{{{E} + \sqrt{{{E}^2} - 4} }}{2}}. \end{aligned}$$

Note that these wavenumbers admit the relation $k_w k_e=1$, as mentioned before.

**Figure 2:**
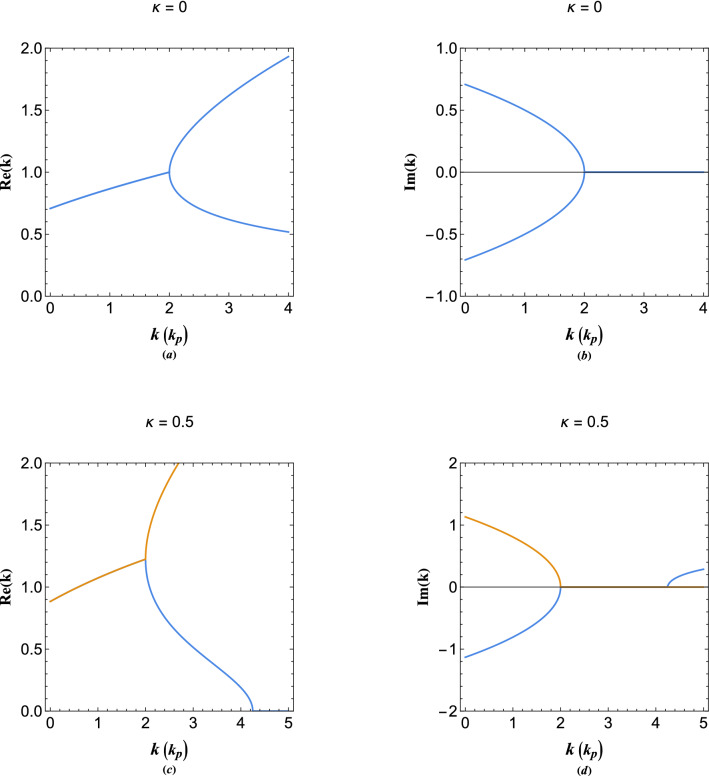
Figures (**a**,**b**), respectively, depict the real and imaginary parts of the energy dispersion of collective quasiparticle modes of undamping plasmon excitations. Figures (**c**,**d**), respectively, depict the real and imaginary parts of the energy dispersion of collective electron modes in the presence of damped plasmon excitations effect ($\kappa =0.5$).

Figure 2a,b depict the real and imaginary parts of the wavenumbers, respectively. The evident branching point at $E=2$ is reminiscent of the exceptional point found in damped harmonic oscillator problem. For a good review of the phenomenon the reader is referred to Ref.^91^. Occurrence of such behavior in Hermitian systems such as current case is outstanding. Recently, it has been shown that such behavior can occur in coupled oscillators with the damping effect replaced by the elastic coupling^93^. The collisionless damping^94,95^ is a well-known character of the wave-particle interactions in electron plasmas. The existence of exception point in the coupled system (5) may be the mathematical manifestation of the kinetic Landau damping effect. Note that the solution (6) does not correspond to the exceptional (singular) point for which $k_w=k_e=k_p$ or equivalently $E=2$. The solution at the exceptional point is found to be

8 $$\begin{aligned} \Phi (x) = \frac{x}{2} \sin (x),\quad \Psi (x) = \cos (x) - \frac{x}{2} \sin (x), \end{aligned}$$

It is remarked that at energies above the exceptional point both wavenumbers are real, whereas, below this point the wavenumbers are complex with the real and imaginary parts being equal but with opposite signs. This means physically that wave-like(particle-like) spacial oscillations grow(decay) for quasiparticle energies $E<2$.

## The pseudo-damped plasmon excitations

Let us now consider the spacial damping effect in non-Hermitian system

9a $$\begin{aligned}&\frac{{{d^2}\Psi (x)}}{{d{x^2}}} + 2\kappa \frac{{d\Psi (x)}}{{dx}} + \Phi (x) + E\Psi (x) = 0, \end{aligned}$$

9b $$\begin{aligned}&\frac{{{d^2}\Phi (x)}}{{d{x^2}}} + 2\kappa \frac{{d\Phi (x)}}{{dx}} - \Psi (x) = 0, \end{aligned}$$

in which the term including the parameter $\kappa$ characterizes the non-Hermiticity and is used to model variety of physical situations^64,65^. The system (9) has the following exact solution for the same boundary values $\Psi (0)=1$ and $\Phi (0)=\Psi '(0)=\Phi '(0)=0$ as before

10a $$\begin{aligned} \Phi (x)&= \frac{{{{{e}}^{ - \kappa x}}}}{{\sqrt{{{E}^2} - 4} }}\left[ {\cos ({k_w}x) - \cos ({k_e}x) + \frac{\kappa }{{{k_w}}}\sin ({k_w}x) - \frac{\kappa }{{{k_e}}}\sin ({k_p}x)} \right] , \end{aligned}$$

10b $$\begin{aligned} \Psi (x)&= \frac{{{{{e}}^{ - \kappa x}}}}{{2\sqrt{{{E}^2} - 4} }}\left[ {\left( {{E} + \sqrt{{{E}^2} - 4} } \right) \cos ({k_p}x) - \left( {{E} - \sqrt{{{E}^2} - 4} } \right) \cos ({k_w}x) } \right. \end{aligned}$$

10c $$\begin{aligned}&\quad + \left. {\frac{{\kappa \left( {{E} + \sqrt{{{E}^2} - 4} } \right) }}{{{k_e}}}\sin ({k_e}x) - \frac{{\kappa \left( {{E} - \sqrt{{{E}^2} - 4} } \right) }}{{{k_w}}}\sin ({k_w}x)} \right] , \end{aligned}$$

with the plasmon oscillation wavenumbers given as

11 $$\begin{aligned} {k_w} = \sqrt{\frac{{{E} - 2\kappa ^2 - \sqrt{{{E}^2} - 4} }}{2}},\quad {k_e} = \sqrt{\frac{{{E} - 2\kappa ^2 + \sqrt{{{E}^2} - 4} }}{2}}. \end{aligned}$$

satisfying the energy dispersion relation $E=(k^2+\kappa ^2)+1/(k^2+\kappa ^2)$. Note that in the limit $\kappa =0$ this reduces to the undamped plasmon dispersion. The product of damped plasmon wavenumbers (11) can be easily shown to satisfy a more general relation, $k_w k_e = \sqrt{\kappa ^4-\kappa ^2 E+1}$. The existence of exponentially decaying term in the solution (9) leads to spacial damping of electron density and electrostatic energy at all energy orbital. The real and imaginary parts of the damped plasmon wavenumbers are shown in Fig. 2c,d for parameter value of $\kappa =0.5$. It is clearly evident that another exceptional point emmerges at $k\simeq 4.25$ in normalized unit beside the original one at $k=1$. Note also that for energy orbital above the new exceptional point the oscilltions behave purely particle-like. It is further revealed that the new exceptional point coincides the orbital energy of $E=\kappa ^2+1/\kappa ^2$. It is obvious that the energy dispersion relation of damped plasmon excitations solely characterize the oscillation types and does not mean an absolute exponential decay on all exceptional point domain. The exceptional behavior of the solution (9) will be discussed in detail in the following section. The solution to the damped system (9) at the exceptional point $E=2$ has the following form

12a $$\begin{aligned} \Phi (x)&-= \frac{{{{{e}}^{ - \kappa x}}}}{{2{{\left( {1 - {\kappa ^2}} \right) }^{3/2}}}}\left\{ {\left[ {\kappa - x\left( {{\kappa ^2} - 1} \right) } \right] \sin \left( {\sqrt{1 - {\kappa ^2}} x} \right) - \kappa \sqrt{1 - {\kappa ^2}} x\cos \left( {\sqrt{1 - {\kappa ^2}} x} \right) } \right\} , \end{aligned}$$

12b $$\begin{aligned} x\Psi (x)&-= \frac{{{{{e}}^{ - \kappa x}}}}{{2{{\left( {1 - {\kappa ^2}} \right) }^{3/2}}}}\left[ {\left( {{\kappa ^2}x + \kappa - 2{\kappa ^3} - x} \right) \sin \left( {\sqrt{1 - {\kappa ^2}} x} \right) } \right. \end{aligned}$$

12c $$\begin{aligned}&\quad - \left. {\sqrt{1 - {\kappa ^2}} \left( {2{\kappa ^2} - \kappa x - 2} \right) \cos \left( {\sqrt{1 - {\kappa ^2}} x} \right) } \right] . \end{aligned}$$

Furthermore, the damped solution at the exceptional point, $E=\kappa ^2+1/\kappa ^2$, reads

13a $$\begin{aligned}&\Phi (x) = \frac{{{\kappa ^2}{{{e}}^{ - \kappa x}}}}{{{{\left( {1 - {\kappa ^4}} \right) }^{3/2}}}}\left[ {\left( {1 + \kappa x} \right) \sqrt{1 - {\kappa ^4}} - \sqrt{1 - {\kappa ^4}} \cos \left( {\frac{{\sqrt{1 - {\kappa ^4}} }}{\kappa }x} \right) - {\kappa ^2}\sin \left( {\frac{{\sqrt{1 - {\kappa ^4}} }}{\kappa }x} \right) } \right] , \end{aligned}$$

13b $$\begin{aligned}&\Psi (x) = \frac{{{{{e}}^{ - \kappa x}}}}{{{{\left( {1 - {\kappa ^4}} \right) }^{3/2}}}}\left[ {\sqrt{1 - {\kappa ^4}} \cos \left( {\frac{{\sqrt{1 - {\kappa ^4}} }}{\kappa }x} \right) + {\kappa ^2}\sin \left( {\frac{{\sqrt{1 - {\kappa ^4}} }}{\kappa }x} \right) - {\kappa ^4}\left( {1 + \kappa x} \right) \sqrt{1 - {\kappa ^4}} } \right] . \end{aligned}$$

**Figure 3:**
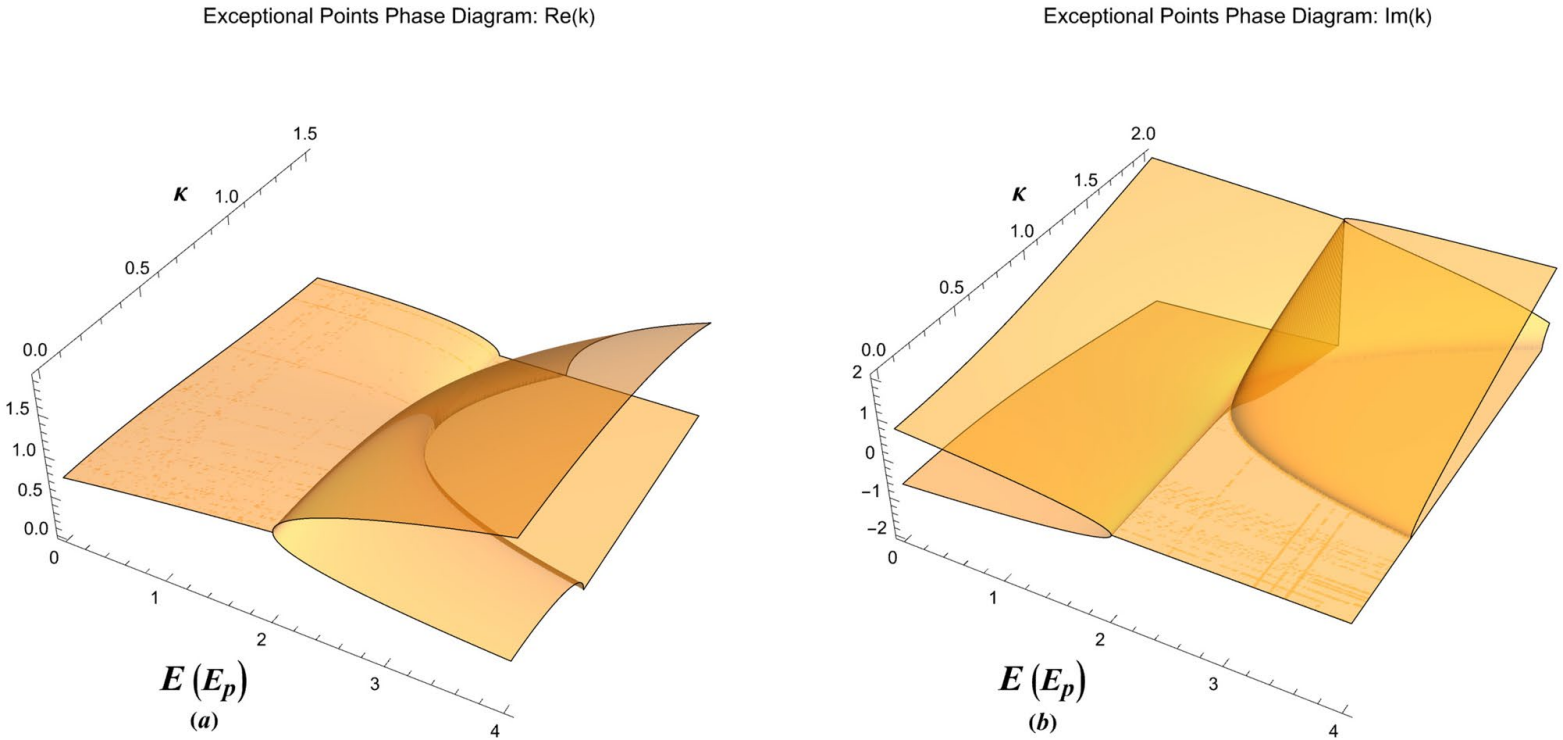
The exceptional point phase diagram of damped oscillations showing (**a**) the real part and (**b**) the imaginary part of the collective oscillation wavenumber in *E*-$\kappa$ plane.

Figure 3 shows a feature-rich 3D exceptional point phase-diagram in *E*-*k* plane. The real/imaginary part of wave-like and particle-like wavenumbers is shown in Fig. 3a,b for undamped collective excitations. The exceptional points phase-diagrams in our case can have a wide variety of physical application in the case of quantum charge screening, light scattering from metallic surfaces and electron spill-out effects. In the following section we show an important application of the damped plasmon model in collective quantum tunneling, the so-called photo-plasmonic effect. We also discuss different exceptional solutions and their physical interpretations in various domains of the exceptional point phase diagram.

## Half-space excitations and photo-plasmonic effect

**Figure 4:**
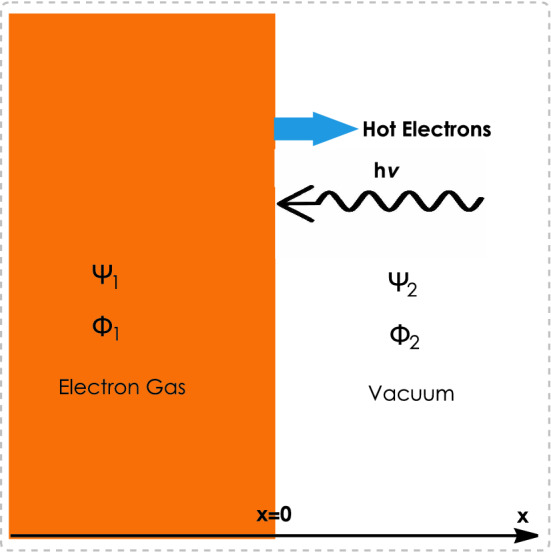
The schematic of collective hot-electron release at the irradiated metal–vacuum interface leading to the energetic photo-plasmonic effect. The wavefunction $\Psi (x)$ and electrostatic energy $\Phi (x)$ characterize the quantum state at each region.

**Figure 5:**
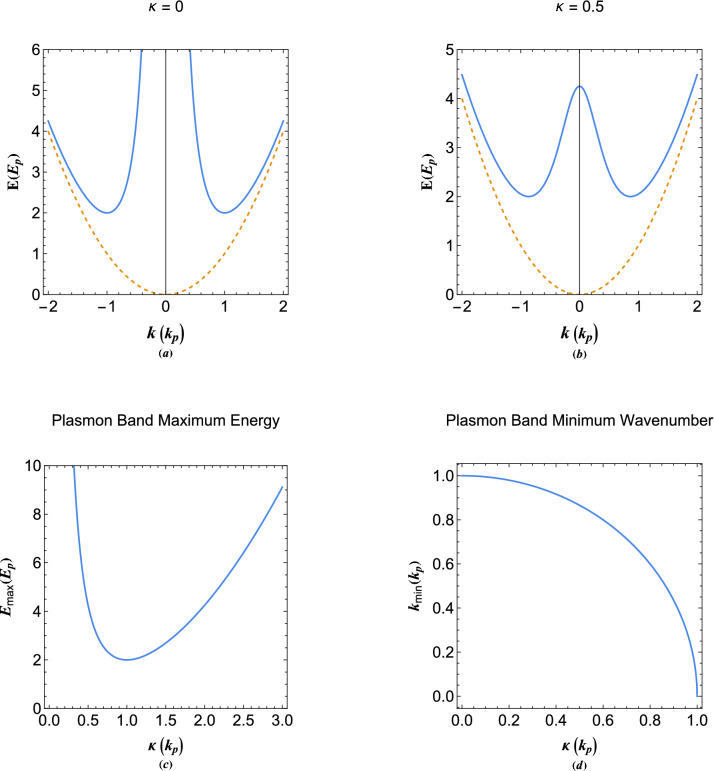
(**a**) The energy dispersion of collective modes in metallic region without damping effect. (**b**) The energy dispersion of damped plasmons in the tunneling region (vacuum) for the given value of the damping parameter ($\kappa =0.5$). (**c**) The maximum energy band values in damped plasmon excitations in Fig. 5b as a function of the damping parameter. (**d**) The minimum energy band wavenumber of damped plasmons (conduction band) as a function of the damping parameter. The dashed curves in plots (**a**,**b**) show the free electron dispersion.

The model (9) has been recently used to describe the electron spill-out effect in half-space configuration^64^ where the parameter $\kappa$ characterizes the collective electron tunneling effect. Therefore, in current half-space excitation model, the undamped solution (6) is used for the metallic side and the damped solution (10) is applied to the vacuum satisfying the boundary conditions at the interface. The mathematical implementation of half-space plasmon excitations are given in Ref.^64^. The damping parameter, $\kappa$, in the independent-electron approximation may be related to the single electron tunneling parameter^64^ via a simple equation, $\kappa =\sqrt{W-E}$, in which $W=V-E_F$ is the work function with *V* and $E_F$ denoting the induced vacuum potential and Fermi energy of metal, respectively. It has been shown^64^ that the nature of collective and single-electron tunneling can be quite different. However, in the case of collective interaction the damping parameter may depend on other dominant phenomena such as the electron–electron scattering and spill-out electron number-density. The development of exact theory for collective tunneling depends on experimental study of the dependence of the electron tunnelling lengthscale on these parameters at the metallic interfaces, using an appropriate tool such as the Langmuir probe. Figure 4 shows the schematic profile of the half-space plasmon excitations and photo-plasmonic effect in which the dashed region ($x<0$) denotes the plasmonic material interfacing the vacuum ($x>0$). It has been shown that^64^ for non-irradiated surface the electron spill-out causes the electron-ion separation and dipole formation at the metal vacuum interface in small region of few angstroms width for a typical metal densities. However, the incident high energy photons can lead to the surface resonance of spill out electrons and the collective generation of hot-electrons from the metallic surface by the photo-plasmonic effect. Therefore, in the following we propose an alternative physical description of the hot electron generation mechanism based on the collective quantum electron tunneling effect.

In the metallic region the collective excitations are stable above the main exceptional point of the system above the orbital, $E=2$. At thermal equilibrium, there are finite number of electrons excited to plasmon band as shown in Fig. 5a. On the other hand, in the vacuum region the spill-out electrons form a narrow unstable plasmon band limited from above at $E=\kappa ^2+1/\kappa ^2$, as shown in Fig. 5b. The variations of maximum energy of the damped plasmon band in vacuum in terms of electron tunneling parameter $\kappa$ is depicted in Fig. 5c showing a minimum at $\kappa =1$, where the two exceptional points coincide. Also, the plasmon band minimum wavenumber varies with the parameter $\kappa$ as shown in Fig. 5d. Note that for $\kappa \ge 1$ there is only a single exceptional point in the system.

**Figure 6:**
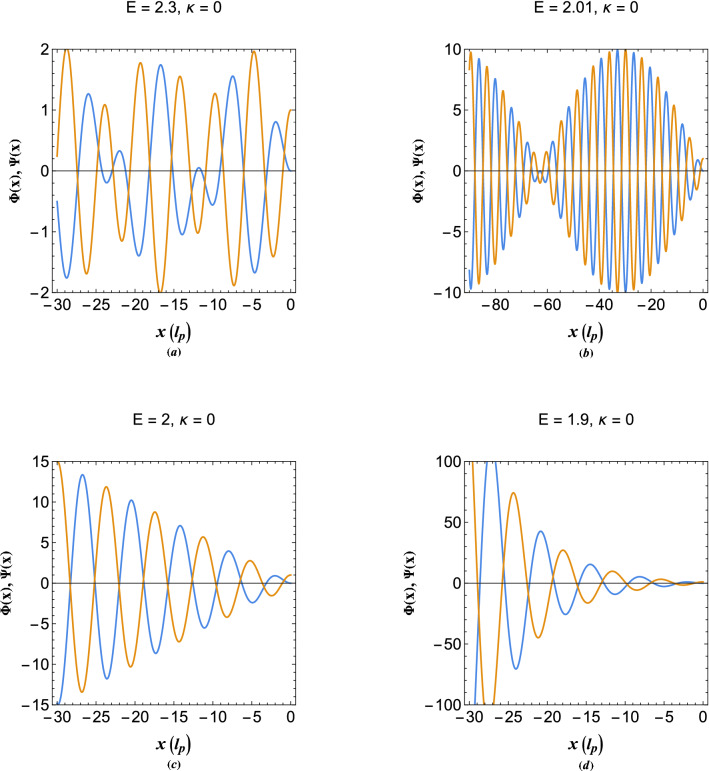
(**a**) The collective oscillation profiles of the local electron density and electrostatic energy of undamped plasmons at overcritical energy orbital (orbitals above the main exceptional point). (**b**) The collective oscillation profiles of the electron density and electrostatic energy of undamped plasmons at nearcritical energy orbital (the quantum beating state). (**c**) The collective oscillation profiles of the local electron density and electrostatic energy of undamped plasmons at exceptional-point energy orbital (orbital at the exceptional point). (**d**) The collective oscillation profiles of the electron density and electrostatic energy of undamped plasmons at undercritical energy orbital (orbitals below the exceptional point in plasmon energy gap region).

Figure 6 depicts the excitation profiles in the metallic region below and above the main exceptional orbital. Figure 6a shows the normalized electron density and electrostatic energy profiles at energy orbital, $E=2.3$, above the main exceptional point. It is remarked that oscillations are of dual-tone nature due to both wave and particle oscillations and are evidently spatially stable. Moreover, Fig. 6b shows the electron density and electrostatic energy profiles at energy orbital close to the main exceptional point, where $k_w\simeq k_e$, which is called the quantum beating orbital. At this orbital the electron density and the electrostatic energy tend to localize and the wave-particle interactions are enhanced quite similar to the quantum interference effect. Figure 6c depicts the solutions below the main exceptional point indicating that the electron density and electrostatic energy grows linearly, hence, the excitations become unstable. Furthermore, in the plasmon band gap region, $0<E<2$, no stable undamped! collective excitations can exist as remarked in Fig. 6d for the energy orbital $E=1.9$ depicting exponential wave-like growth.

**Figure 7:**
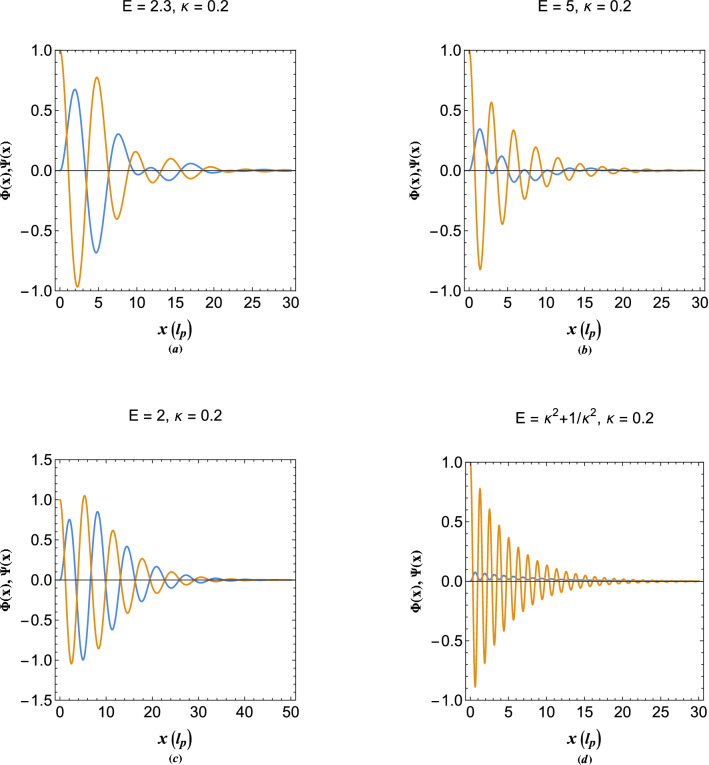
(**a**) The collective oscillation profiles of the local electron density and electrostatic energy of damped plasmons at energy orbital between the two exceptionl points. (**b**) The collective oscillation profiles of the local electron density and electrostatic energy of damped plasmons at energy orbital above the two exceptional points. (**c**) The collective oscillation profiles of the local electron density and electrostatic energy of damped plasmons at exceptional-point energy orbital (orbital at the main exceptional point $E=2$). (**d**) The collective oscillation profiles of the local electron density and electrostatic energy of damped plasmons at second exceptional point energy orbital (orbital at the second exceptional point with the energy, $E=\kappa ^2+1/\kappa ^2$).

The excitations of tunneling (spill out) electrons in vacuum follows the solution (10). Figure 7 shows such excitations in different parametric regions in vacuum side. Figure 7a shows the excitation profiles for energy orbital between the two exceptional points. It is seen that the oscillations are strongly damped in vacuum region at distances away from the interface, due to the decay character of quantum electron tunneling. However, the plasmon damping in this region is double-tone because of wave and particle excitations which distinguishes it from single electron case. Figure 7b depicts the excitation profiles for orbital above the two exceptional point, indicating that collective excitations are damped also in this region. Figure 7c,d show the excitation profiles at the exceptional point $E=2$ and $E=\kappa ^2+1/\kappa ^2$, respectively. While the amplitude of oscillations in denisty and energy at orbital $E=2$ of Fig. 7c are nearly the same as shown in Fig. 7a,b, the amplitude of energy oscillations is much lower compared to the density at the second exceptional point shown in Fig. 7d. It is also remarked that at the exceptional points the oscillations are single tone due to coincidence of real and imaginary wavenumbers at such points.

**Figure 8:**
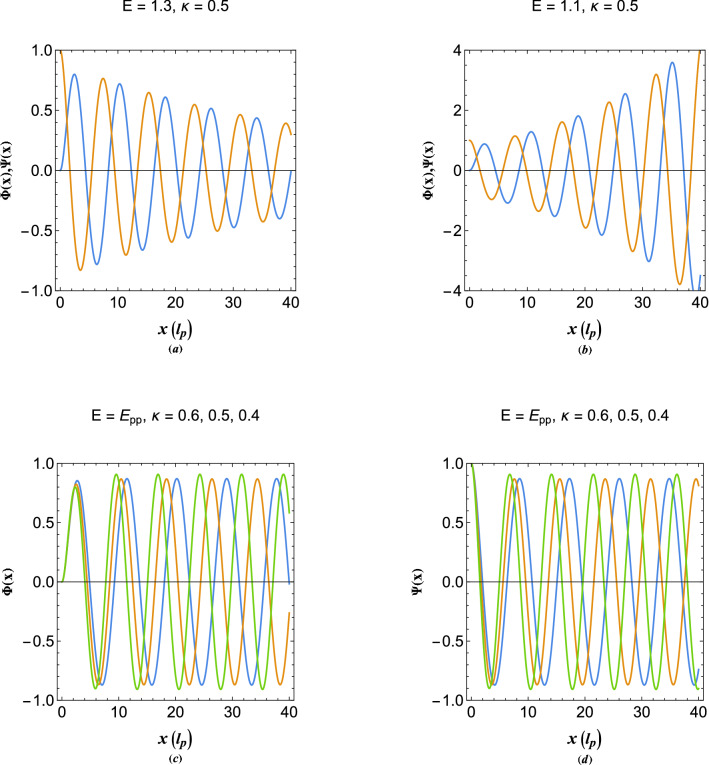
(**a**) The decaying unstable collective oscillation profiles of the local electron density and electrostatic energy of dampes plasmons at energy orbital below the main exceptionl point, $E=2$. (**b**) The growing unstable collective oscillation profiles of the local electron density and electrostatic energy of damped plasmons at energy orbital below the main exceptional point, $E=2$. (**c**) The stable collective oscillation profiles of the electrostatic energy of damped plasmons at resonant orbital below the main exceptionl point, $E=2$. (**d**) The stable collective oscillation profiles of the electron density of damped plasmons at resonant orbital below the exceptionl point, $E=2$.

Figure 8a,b show the damped excitation profiles in the plasmon band gap below the main exceptional point in vacuum. It is shown that the excitations can be of both decaying and growing type depending on orbital energy. However, both oscillation types in Fig. 8a,b are unstable. It is found that there exist a critical orbital in this region at which the excitations become stable. This becomes possible, however, due to the resonant matching of the real (damping) particle-like oscillation wavenumber with that of the imaginary (growing) wave-like oscillations. This phenomenon is a collective assistance in electron tunnelling which otherwise would rapidly decay. It is remarked that for each value of the tunneling parameter there is a distinct resonant orbital, here termed as the photo-plasmon orbital, $E_{pp}$, for which the excitation become stable in the vacuum. This is a novel feature of dual-tone plasmon excitations in damping environment which takes place solely due to multiple character of collective electrostatic excitations. The photo-plasmonic effect is the plasmon-assisted hot-electron tunneling and it may be considered as the collective tunneling analogous of the well-know photo-electric effect. From Fig. 8c,d, it is clearly remarked that, while the wavenumber of oscillations at the photo-plasmonic orbital $E=\{1.02447,1.23607,1.4599\}$ corresponding, respectively, to damping values $\kappa =\{0.6,0.5,0.4\}$ strongly varies with the changes in value of the damping parameter, $\kappa$, the oscillation amplitude of electron density and electrostatic energy in vacuum side is almost independent of this parameter and becomes plane-wave type in distances away from the interface. In reality energetic (hot) electrons can be either excited directly to the photo-plasmonic orbital by appropriate radiation at the metallic surface or the electrons excited to plasmon conduction band damp through a lossless Landau-like mechanism and collectively tunnel through the vacuum potential barrier which is an indirect mechanism involving a change in the momentum. In current research we assume the later mechanism as the feasible photo-plasmonic effect. The photo-plasmonic effect can have profound applications in solar-cell technology and effective photo-electron energy extraction. Current solar cell designs rely on inefficient electron–hole pair excitations at the Fermi level of semiconductor junctions. The resonant photo-plasmon energy orbital, $E_{pp}$, can be obtained from the following wavenumber-matching condition

14 $$\begin{aligned} \Im \left( {\sqrt{{E_{pp}} - 2{\kappa ^2} - \sqrt{{{E_{pp}}^2} - 4} } } \right) + \sqrt{2} \kappa = 0, \end{aligned}$$

where $\Im$ denotes the imaginary part of the complex variable. The generalized wavenumbers of damped plasmon excitations (including the exponential damping term) may be written as

15 $$\begin{aligned} {K_w^\pm } = - \kappa \pm {{i}}\sqrt{\frac{{{E} - 2{\kappa ^2} - \sqrt{{{E}^2} - 4} }}{2}},\quad {K_e^\pm } = - \kappa \pm {{i}}\sqrt{\frac{{{E} - 2{\kappa ^2} + \sqrt{{{E}^2} - 4} }}{2}}. \end{aligned}$$

**Figure 9:**
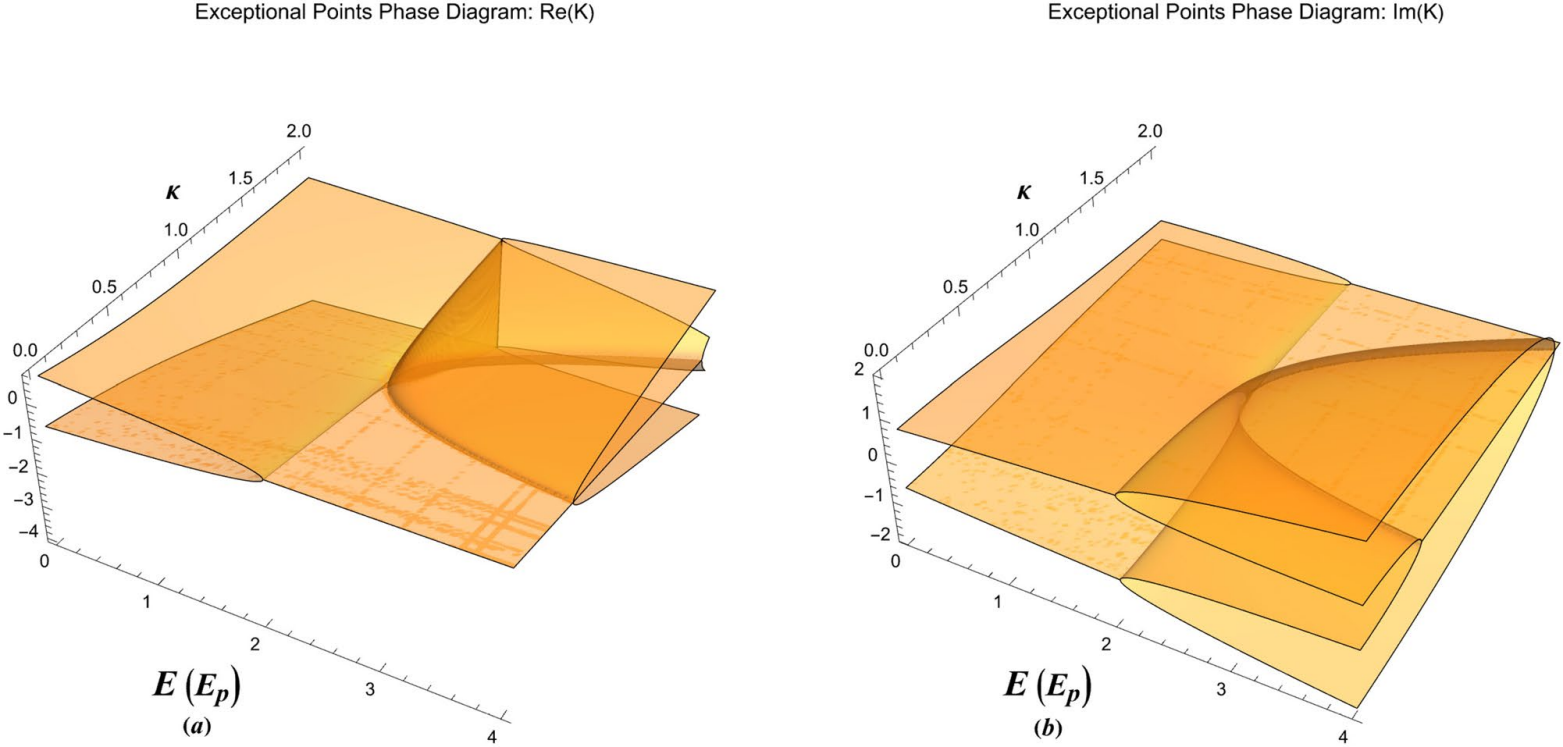
(**a**) The real part of generalized damped plasmon wavenubers. (**b**) The imaginary part of generalized damped plasmon wavenubers.

The photo-plasmon tunneling (surface resonance) condition is then briefly stated as, $\Re (K_w^+)=\Re (K_e^-)=0$, where $\Re$ denotes the real part of a variable. It is also noted that the relation, $\Im (K_w^\pm )=\Im (K_e^\mp )$, always holds. Note also that the later exceptional resonant condition is simply obtained by matching the generalized damped plasmon wavenumbers (15), so that the wavefunction solution in the vacuum side, away from the interface, becomes of plane-wave type which in turn is interpreted as the collective quantum tunneling of spill-out electrons. The exceptional point phase diagram corresponding to the generalized wavenumbers, *K*, is shown in Fig. 9. It is remarked that the structure of exceptional diagram in the plasmon tunneling region is much more complex as compared to that of Fig. 3. This makes possible existence of variety of other novel physical phenomenon with possibly important technological applications in the collective quantum electron behavior and photo-electron interaction effects via fine tuning of the the photon energy and the quantum damping parameter.

**Figure 10:**
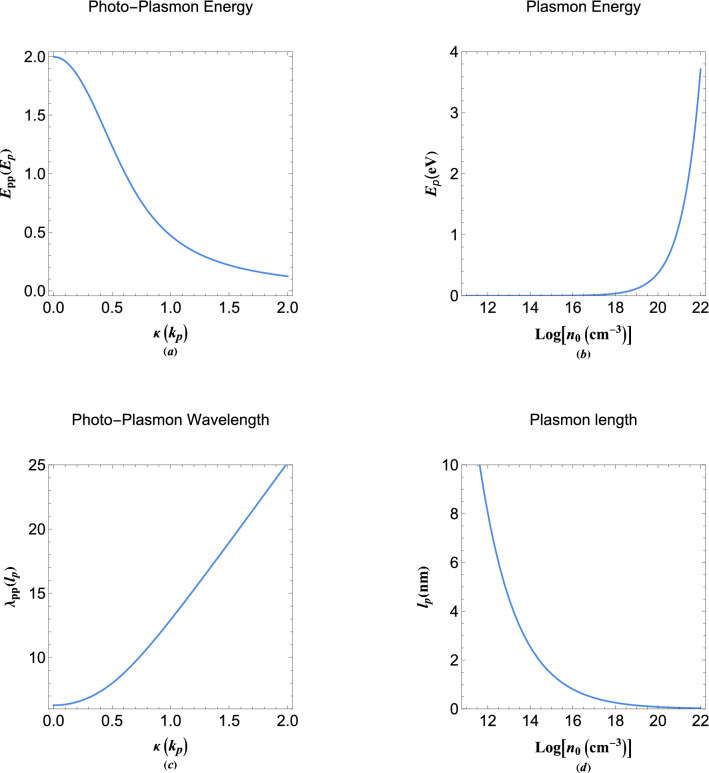
(**a**) Variation of photo-plasmon energy with the damping parameter which is related to the collective tunneling parameter in the plasmon units. (**b**) The variation in plasmon energy unit with electron number-density. (**c**) The variation of photo-plasmon wavelength with respect to the damping parameter. (**d**) The variation of plasmon length in nanometer unit with the electron density.

In Fig. 10a, we have shown the photo-plasmon energy variation with the damping parameter in plasmon units. It is remarked that the photo-plasmon energy reduces by increase in the damping parameter which directly related to the metal–vacuum interface quantum electron tunneling phenomenon. The maximum energy is seen to correspond to the undamped case. Technically the hot-electron extraction may include some efficient tandem multilayer material design for best performance. In order to compare the energy scale in plasmonic devices we have shown the variation of the plasmon energy with the free electron concentration in Fig. 10b. The values of this energy for typical metallic densities are few electronvolts. For the aluminium as a good plasmonic material candidate this energy can be as high as $E_p^{Al}\simeq 15$ eV. Figure 10c depicts the variation in the photo-plasmon wavelength in plasmon length ($l_p=1/k_p$) units. It is clearly evident that by increase in the damping parameter the photo-plasmon wavelength increases sharply. The variation of plasmon length with electron number density is shown in Fig. 10d in nanometer unit. For typical metals the photo-plasmon wavelength is around few nanometers for a typical value of the damping parameter.

Current model is based on the generalized matter-wave dispersion of collective quasiparticle excitations and can provide even useful information on electronic density and internal energy distribution on the quasiequilibrium vacuum region by replacing the undamped energy dispersion with the general damped dispersion relation in Eq. (4) and calculating the DoS of damped excitation. However, due to the local variation of the electron number density and chemical potential in the vacuum side, one needs to know the spill-out electron distribution which can obtained by statistical averaging the wavefunction as described in Ref.^64^.

**Figure 11:**
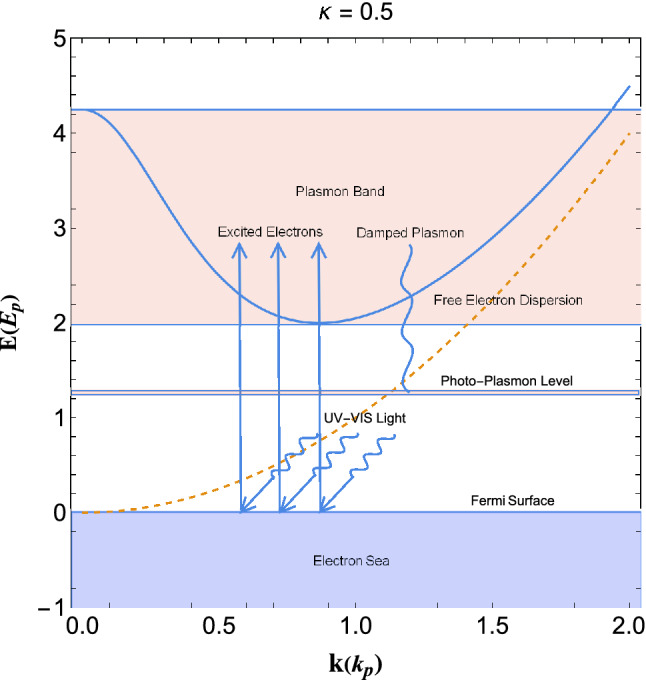
Schematic of the photo-plamonic phenomenon in the arbitrary degenerate electron gas at the interface region. The dashed curve represents the free electron dispersion and the solid curve denotes the damped plasmon dispersion.

Figure 11 shows the schematic of photo-plasmonic effect at the irradiated surface of plasmonic metal with $\kappa =0.5$. The dashed curve depicts the free electron dispersion and the solid curve denotes the damped plasmon energy dispersion. The zero orbital point, $E=0$, denotes the Fermi energy level below which electrons are packed at zero temperature valence band. It is remarked that electrons at the Fermi surface of the metal can be excited to the plasmon band by energetic visible or ultraviolet radiations. They either directly excite to the resonant orbital or excite to stirring plasmon band and then collectively damp and fall into the photo-plasmon energy level from which they collectively tunnel through vacuum or semiconductor interface. Note that the collective excitation behavior of damped plasmons is the main reason for the formation of the photo-plasmon level and consequent hot-electron generation. Also, note that electrons excited beyond the depicted damped plasmon band lose their wave-like behavior and therefore can not contribute to the photo-plasmonic effect via lossless damping. The plasmon band however play the role of a collective stirring band to form energetic electrons collection quite similar in nature to the starling murmuration phenomenon.

## Conclusion

In this research, based on the effective damped Schrödinger–Poisson model, we proposed a novel collective hot-electron generation mechanism at metal–vacuum interfaces. We studied the exceptional behavior of collective excitations from the energy dispersion relation for both undamped and damped coupled differential equations revealing a full-featured exceptional point phase structure in plasmon excitations. Such exceptional behavior are of fundamental importance and technological applications in many branches of physical sciences. We showed that a similar mechanism as the photo-electric effect (the so-called photo-plasmonic effect) exists for the collective excitations at the metal surfaces illuminated by appropriate radiations. The high energy photo-plasmonic electrons may be used in highly efficient plasmonic solar-cell devices for energy harvesting purposes. The photo-plasmonic effect is also physically important phenomenon in understanding the collective quantum electron tunneling and electron spill-out effect in metallic surfaces and metal–semiconductor interfaces.

## Data Availability

The data that support the findings of this study are available from the corresponding author upon reasonable request.
